# Province-specific smoking-attributable cancer mortality in China 2013

**DOI:** 10.18332/tid/122013

**Published:** 2020-06-01

**Authors:** Linjie Yu, Junxia Cheng, Xiaoli Cui, Jianbing Wang

**Affiliations:** 1 Department of Epidemiology and Biostatistics, School of Public Health, Zhejiang University, Hangzhou, People’s Republic of China; 2 Department of Gynecologic Oncology, Cancer Hospital of China Medical University, Liaoning Cancer Hospital & Institute, Shenyang, People’s Republic of China; 3 Department of Epidemiology and Biostatistics, The Children’s Hospital, National Clinical Research Center for Child Health, Zhejiang University School of Medicine, Hangzhou, People’s Republic of China

**Keywords:** smoking, cancer, population attributable fraction, China

## Abstract

**INTRODUCTION:**

Province-specific initiatives are at the forefront of tobacco control but limited studies have provided province-specific assessment of smoking-attributable cancer burden in China.

**METHODS:**

We estimated the fraction of total and site-specific cancer mortality attributable to tobacco smoking in 31 provinces in mainland China. The population attributable fractions (PAFs) for cancer deaths due to smoking were calculated by Levin’s formula using province-specific smoking prevalence data around 1998 (assuming a 15-year latency time) and relative risks from cohort studies and meta-analyses. The 95% confidence intervals (CIs) of PAFs were calculated by a Delta method. Cancer deaths were abstracted from cancer registry data of the 31 provinces in mainland China in 2013.

**RESULTS:**

Overall, smoking contributed to a total of 421566 cancer deaths in mainland China in 2013 (19.46% of all cancer deaths), with 400701 of these deaths occurring in men (29.34%) and 20865 (2.61%) in women. The population attributable fractions ranged from 15.56% (95% CI: 9.12–21.82%) in Tibet to 35.09% (95% CI: 25.68–45.83%) in Guizhou among men, and from 0.28% (95% CI: 0.00–0.64%) in Hainan to 10.44% (95% CI: 4.86–16.32%) in Jilin among women. Cancers of lung and liver were the two main smoking-attributable cancers for both men and women.

**CONCLUSIONS:**

Tobacco smoking was responsible for nearly 20% of all cancer deaths in mainland China, but the proportion of cancer deaths attributable to smoking varied substantially across provinces. More effective programs and innovative new strategies for local tobacco control are warranted to reduce the future burden of smoking-related cancers in all provinces of mainland China.

## INTRODUCTION

Cancer has become a leading cause of death in China in recent decades^1^. A number of epidemiology studies have demonstrated that most cancers are attributable to a modifiable lifestyle and environmental risk factors, among which smoking is the largest preventable one^2–5^. Smoking, with a long-term adverse impact on health, has taken a great toll, of about 1 million deaths per year, on China and this is expected to exceed 3 million in 2050^6^. China is the world’s largest producer and consumer of tobacco, with a population of 1.4 billion it has more than 301 million current smokers^7^. Due to substantial geographical variation in smoking prevalence, the burden of smoking related cancer across provinces remains unclear. In a previous study, we reported for the first time a systematic evaluation of the number of cancer cases and deaths attributable to carcinogens in 2005 in China^8^. In that study, we calculated the cancer cases and deaths attributable to smoking at the national level, but not at the provincial level. It is valuable to assess the local cancer burden caused by tobacco use as province-level initiatives are at the forefront of tobacco control, as indicated in the Lortet-Tieulent et al.^9^ study. However, most studies estimating smoking-related cancer mortality have been at the national level, and limited studies exist at the provincial level^10–13^.

Here, we provide an additional systematic assessment of cancer deaths attributable to smoking at the provincial level in China in 2013, aiming to provide scientific evidence for local policy makers to take effective action in tobacco control and cancer prevention.

## METHODS

### Overview

We estimate the province-specific proportion of cigarette smoking-related cancer mortality using similar methods in previous reports^8,10^. We selected 10 cancers caused by cigarette smoking that have been classified established targets of the carcinogenicity of tobacco by the International Agency for Research on Cancer (IARC) including: cancers of mouth/pharynx/larynx, nasopharynx, lung, stomach, liver, esophagus, pancreas, colorectum, bladder, and kidney.

### Cancer deaths

Data on cancer deaths in China in 2013 were based on the 255 qualified cancer registries distributed in the 31 provinces (autonomous regions and municipalities) of the annual cancer report of National Cancer Center, covering 226494490 of the population (including 114860339 males and 111634151 females) that accounted for 16.65% of the national population in 2013. In each province, cancer death rates were calculated by age group, sex, and cancer site. The death rates with sex-specific, age-specific and province-specific populations in 2013 were extrapolated to estimate the number of cancer deaths in each province. Finally, cancer death cases were calculated by summing the cases across all age groups and cancer sites in each province. This study is based on previously published data and does not include new human data that require again ethical approval and consent. The authors assume that the data source studies were conducted after ethical approval and consent, and in accordance with the Declaration of Helsinki 1975. The authors can confirm that all relevant data are included in the article and materials are available on request from the corresponding author.

### Latency time and smoking prevalence

The occurrence of current cancers always reflects the past patterns of sustained smoking exposure, therefore there is a latency period between exposure and cancer. To date, no accurate studies are available to define the specific latency time between smoking and cancers. A report of World Health Organization (WHO) in 2000 suggested a latency time of 15 years^14^. Therefore, we used the smoking prevalence data of 1998.

Our study used a linear interpolation method to estimate provincial smoking prevalence in 1998 of data from two national surveys, in 1996 and 2002^15,16^, based on the previous study^10^. In our study, smoking was identified as ‘continuous or cumulative smoking of at least one cigarette every day for 6 months or more during the lifetime’^17^. Accordingly, we used the overall smoking status to represent smoking exposure, irrespective of current or former smoking status, type, amount, and duration of smoking.

### Relative risk

Data on relative risks (RRs) were obtained from different sources, including PubMed and China National Knowledge Infrastructure (CNKI), in Chinese or English. High-quality meta-analyses or large-scale pooled analyses from the Chinese population were given the highest priority for RRs between smoking and specific types of cancer mortality, followed by meta-analyses from Asian populations. For most cancers associated with smoking, the RRs used in our study were obtained from a large-scale pooled analysis of smoking and cancer in populations of China and South Korea^18^. However, RR for smoking and kidney cancer mortality in men was from a meta-analysis among the Asian population^19^, and RR for smoking and esophageal cancer in women was abstracted from a prospective study in Linxian, China^20^ (Table 1).

**Table 1:** Relative risks of site-specific smoking-related cancers in China 2013

*Cancer site*	*ICD-10 code*	*Study^a^*	*Design*	*Age (years)*	*RR (95% CI)*	
					*Men*	*Women*
Mouth/larynx/	C00-C10	[18]	Pooled-analysis	≥45	1.95 (1.51–2.5)	1.99 (1.11–3.59)
Pharynx	C12-C14					
Stomach	C15	[18]	Pooled-analysis	≥45	1.43 (1.24–1.64)	1.14 (1.08–1.52)
Colorectal	C18-C20	[18]	Pooled-analysis	≥45	1.13 (0.93–1.37)	1.40 (1.08–1.83)
Liver	C22	[18]	Pooled-analysis	≥45	1.35 (1.19–1.53)	1.75 (1.05–2.84)
Pancreas	C25	[18]	Pooled-analysis	≥45	1.18 (0.75–1.86)	1.65 (1.08–2.53)
Lung	C33-C34	[18]	Pooled-analysis	≥45	3.56 (2.45–5.16)	3.34 (2.29–4.86)
Bladder	C67	[18]	Pooled-analysis	≥45	1.97 (1.26–3.06)	1.41 (0.56–3.52)
Kidney^b^	C64	[19]	Meta-analysis	-	1.11 (0.85–1.47)	1.11 (0.85–1.47)
Nasopharynx	C11	[18]	Pooled-analysis	≥45	2.22 (1.42–3.49)	2.22 (1.42–3.49)
Esophagus	C15	[18]	Pooled-analysis	≥45	1.54 (0.66–3.57)	-
		[20]	Prospective study	40–69	-	1.34 (1.16–1.54)^c^

a Zheng et al.^18^, Cumberbatch et al.^19^ and Tran et al.^20^.

b RR was derived from the Asian population.

c RR for female esophageal cancer associated with smoking was obtained from a prospective study in Linxian, China^20^.

### Statistical analysis

Population attributable fraction (PAF) is defined as the proportional reduction of disease incidence or mortality in a population that would occur if exposure to a risk factor is reduced to an alternative ideal exposure scenario (e.g. no tobacco use)^21^. PAF was calculated according to Levin’s formula, in which p represents the prevalence of exposure to the risk factor in the total population, and RR represents the relative risk of a risk factor, where:

$$\text{PAF=}\frac{\text{p}∙\left(\text{RR-1}\right)}{\text{p}∙\left(\text{RR-1}\right)\text{+1}}$$

The 95% confidence intervals (CIs) of PAF were calculated by a Delta method^22^, assuming that ln(RR) has a normal distribution:

$$\text{var}\left(\text{PAF}\right)\text{=}\frac{{\left(\text{RR-1}\right)}^{\text{2}}∙\text{var}\left(\text{p}\right)\text{+}{\left(\text{p}∙\text{RR}\right)}^{\text{2}}∙\text{var}\left[\text{ln}\left(\text{RR}\right)\right]}{{\left[\text{p}∙\left(\text{RR-1}\right)\text{+1}\right]}^{\text{4}}}$$

Finally, the overall PAF in each province was calculated by dividing the number of estimated smoking-attributable cancer deaths by the total number of cancer deaths among persons aged ≥30 years, in each province.

## RESULTS

In 2013, smoking contributed to a total of 421566 cancer deaths (19.46% of all cancer deaths) in mainland China, with 400701 of these deaths occurring in men (29.34%) and 21368 (2.67%) in women (Table 2).

**Table 2:** Population (N), smoking prevalence (%), cancer deaths (CD), number and proportion of smoking-attributable cancer deaths (SACD), China 2013

*Region*	*Province*	*N*	*%*	*CD*	*SACD*	*PAF % (95% CI)*	*PAF rank*
**Men**							
**Northern**	Beijing	10126430	66.82	20426	5967	29.21 (19.89–41.19)	16
	Tianjin	6907091	59.71	13046	4066	31.17 (21.85–42.44)	6
	Hebei	36430286	63.41	65439	18165	27.76 (18.25–39.88)	22
	Shanxi	18338760	64.97	33416	9968	29.83 (18.79–44.60)	13
	Inner Mongolia	12838243	69.28	24398	7494	30.72 (19.79–45.18)	7
**Northeast**	Liaoning	22147745	54.70	53673	15198	28.32 (18.72–40.34)	21
	Jilin	13907218	64.39	24847	7863	31.65 (22.20–42.76)	4
	Heilongjiang	19426106	59.76	43269	13173	30.44 (21.05–42.01)	9
**Eastern**	Shanghai	11854916	64.90	38578	9949	25.79 (17.07–37.01)	26
	Jiangsu	39626707	61.78	79512	21920	27.57 (16.66–42.67)	23
	Zhejiang	27965641	63.22	65728	20006	30.44 (20.72–42.70)	9
	Anhui	30245513	62.02	67127	20583	30.66 (19.11–45.71)	8
	Fujian	18981054	61.47	41173	10898	26.47 (16.21–40.01)	24
	Jiangxi	23003521	62.96	37340	10922	29.25 (19.99–40.20)	15
	Shandong	48446944	55.78	119352	34230	28.68 (18.27–42.41)	18
**Central**	Henan	47493063	63.10	90311	26199	29.01 (17.95–43.82)	17
	Hubei	29391247	65.03	64673	20533	31.75 (21.88–43.75)	3
	Hunan	33776459	63.72	52206	16417	31.45 (22.40–41.67)	5
**Southern**	Guangdong	54400538	66.04	92992	28205	30.33 (20.67–41.82)	12
	Guangxi	23924704	57.54	47584	13515	28.40 (19.20–39.04)	19
	Hainan	4592283	54.30	8876	2112	23.79 (15.63–32.88)	30
**Southwest**	Chongqing	14608870	66.66	34925	12164	34.83 (23.38–49.80)	2
	Sichuan	40827834	63.38	95297	28125	29.51 (18.48–44.24)	14
	Guizhou	17905471	73.82	23344	8191	35.09 (25.68–45.83)	1
	Yunnan	23856696	75.48	35566	10803	30.37 (21.41–41.41)	11
	Tibet	1542652	47.67	559	87	15.56 (9.12–21.82)	31
**Northwest**	Shaanxi	19287575	63.50	35344	10014	28.33 (16.88–44.28)	20
	Gansu	13064193	62.76	32955	7968	24.18 (13.48–38.87)	29
	Qinghai	2913793	67.45	4078	1048	25.70 (16.14–37.96)	27
	Ningxia	3227404	59.38	4602	1213	26.36 (17.08–38.16)	25
	Xinjiang	11270147	50.36	14917	3705	24.84 (16.06–36.03)	28
	National	682329104		1365553	400701	29.34 (19.21–42.31)	
**Women**							
**Northern**	Beijing	9485938	6.93	14279	670	4.69 (1.90–7.71)	5
	Tianjin	6031602	11.56	9599	901	9.39 (4.49–14.52)	2
	Hebei	35423924	5.55	39607	1431	3.61 (1.51–5.85)	6
	Shanxi	17373341	2.56	18375	285	1.55 (0.55–2.67)	21
	Inner Mongolia	11868048	12.50	12667	1182	9.33 (4.19–14.77)	3
**Northeast**	Liaoning	21598578	5.45	47353	1550	3.27 (1.29–5.35)	8
	Jilin	13545597	14.91	16577	1730	10.44 (4.86–16.32)	1
	Heilongjiang	18887885	10.67	29662	2544	8.58 (3.78–13.64)	4
**Eastern**	Shanghai	11164280	2.09	27631	324	1.17 (0.29–2.15)	25
	Jiangsu	39034234	3.53	45097	1002	2.22 (0.81–3.75)	13
	Zhejiang	26461250	1.90	35320	481	1.36 (0.42–2.37)	24
	Anhui	29254955	4.08	33289	811	2.44 (0.89–4.13)	10
	Fujian	17913163	0.82	18249	89	0.49 (0.10–0.91)	30
	Jiangxi	21564276	2.42	19975	308	1.54 (0.48–2.67)	22
	Shandong	47345775	2.73	66521	1448	2.18 (0.85–3.58)	14
**Central**	Henan	46536876	1.39	61735	549	0.89 (0.29–1.53)	28
	Hubei	27846480	2.71	33938	651	1.92 (0.67–3.29)	18
	Hunan	31924303	3.11	28374	575	2.03 (0.72–3.43)	16
**Southern**	Guangdong	49919921	2.54	53371	1023	1.92 (0.62–3.28)	18
	Guangxi	22099057	2.03	22450	331	1.47 (0.46–2.57)	23
	Hainan	4079202	0.53	4657	13	0.28 (0.00–0.64)	31
**Southwest**	Chongqing	14237300	2.88	18652	428	2.29 (0.88–3.80)	12
	Sichuan	39589694	2.77	49805	974	1.96 (0.69–3.30)	17
	Guizhou	16843085	2.76	14640	340	2.32 (0.75–4.00)	11
	Yunnan	22110070	3.65	24683	504	2.04 (0.66–3.52)	15
	Tibet	1459513	6.97	399	4	1.00 (0.25–2.01)	27
**Northwest**	Shaanxi	18039804	1.94	19929	222	1.11 (0.39–1.90)	26
	Gansu	12511070	1.67	19858	151	0.76 (0.24–1.36)	29
	Qinghai	2712930	2.90	2366	37	1.56 (0.42–2.92)	20
	Ningxia	3073946	4.93	2536	84	3.31 (1.26–5.60)	7
	Xinjiang	10545668	3.85	8993	223	2.48 (0.88–4.24)	9
	National	650481765		800587	20865	2.61 (1.01–4.31)	
**Total**							
**Northern**	Beijing	19612368	37.85	34705	6637	19.12 (12.49–27.41)	17
	Tianjin	12938693	37.26	22645	4967	21.93 (14.49–30.61)	5
	Hebei	71854210	34.88	105046	19596	18.65 (11.94–27.05)	19
	Shanxi	35712101	34.61	51791	10253	19.80 (12.32–29.72)	13
	Inner Mongolia	24706291	42.00	37065	8676	23.41 (14.46–34.79)	2
**Northeast**	Liaoning	43746323	30.39	101026	16748	16.58 (10.55–23.94)	26
	Jilin	27452815	39.97	41424	9593	23.16 (15.26–32.18)	3
	Heilongjiang	38313991	35.56	72931	15717	21.55 (14.03–30.47)	6
**Eastern**	Shanghai	23019196	34.43	66209	10273	15.52 (10.07–22.46)	29
	Jiangsu	78660941	32.88	124609	22922	18.40 (10.92–28.58)	22
	Zhejiang	54426891	33.41	101048	20487	20.27 (13.62–28.60)	10
	Anhui	59500468	33.54	100416	21394	21.31 (13.07–31.93)	8
	Fujian	36894217	32.02	59422	10987	18.49 (11.26–28.00)	21
	Jiangxi	44567797	33.66	57315	11230	19.59 (13.19–27.12)	15
	Shandong	95792719	29.56	185873	35678	19.19 (12.03–28.52)	16
**Central**	Henan	94029939	32.56	152046	26748	17.59 (10.78–26.65)	24
	Hubei	57237727	34.71	98611	21184	21.48 (14.58–29.83)	7
	Hunan	65700762	34.27	80580	16992	21.09 (14.77–28.21)	9
**Southern**	Guangdong	104320459	35.65	146363	29228	19.97 (13.36–27.77)	12
	Guangxi	46023761	30.89	70034	13846	19.77 (13.20–27.35)	14
	Hainan	8671485	28.07	13533	2125	15.70 (10.25–21.78)	28
**Southwest**	Chongqing	28846170	35.83	53577	12592	23.50 (15.55–33.79)	1
	Sichuan	80417528	34.87	145102	29099	20.05 (12.37–30.19)	11
	Guizhou	34748556	38.75	37984	8531	22.46 (16.07–29.71)	4
	Yunnan	45966766	40.12	60249	11307	18.77 (12.91–25.89)	18
	Tibet	3002165	27.94	958	91	9.50 (5.43–13.57)	31
**Northwest**	Shaanxi	37327379	33.89	55273	10236	18.52 (10.93–29.00)	20
	Gansu	25575263	33.06	52813	8119	15.37 (8.50–24.77)	30
	Qinghai	5626723	36.25	6444	1085	16.84 (10.37–25.09)	25
	Ningxia	6301350	32.74	7138	1297	18.17 (11.46–26.59)	23
	Xinjiang	21815815	27.93	23910	3928	16.43 (10.35–24.07)	27
	National	1332810869		2166140	421566	19.46 (12.49–28.26)	

CI: confidence interval. PAF: population attributable fraction. SACD: smoking attributable cancer deaths.

**Figure 1:**
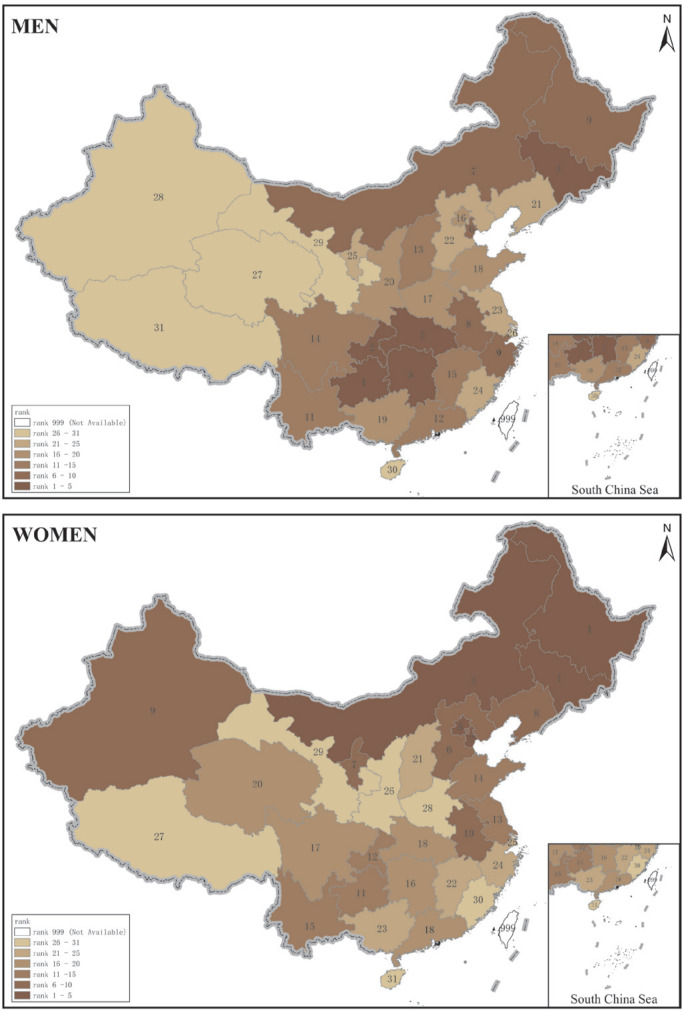
Rank of smoking-attributable cancer mortality in 2013 by gender

For the sexes combined, the faction of cancer deaths attributable to smoking was highest in Inner Mongolia, Chongqing, Jilin and Guizhou, and PAF ranged from 22.46% to 23.50% (Table 2; and Supplementary file, Figure S1). However, the patterns of PAFs were different between men and women, ranging from 15.56% (95% CI: 9.12–21.82%) in Tibet to 35.09% (95% CI: 25.68–45.83%) in Guizhou among men, and from 0.28% (95% CI: 0.00–0.64%) in Hainan to 10.44% (95% CI: 4.86–16.32%) in Jilin among women (Table 2). In men, the top 4 PAFs ranged from 35.09% to 31.65%, for provinces in the Southwest and Northeast regions including Guizhou, Chongqing, Hubei, and Jilin. In women, however, the topmost 4 provinces (Jilin, Tianjin, Inner Mongolia, and Heilongjiang) were located in Northern China, and PAF ranged from 8.58% to 10.44%, which was three-fold of the national average (Figure 1).

**Figure 2:**
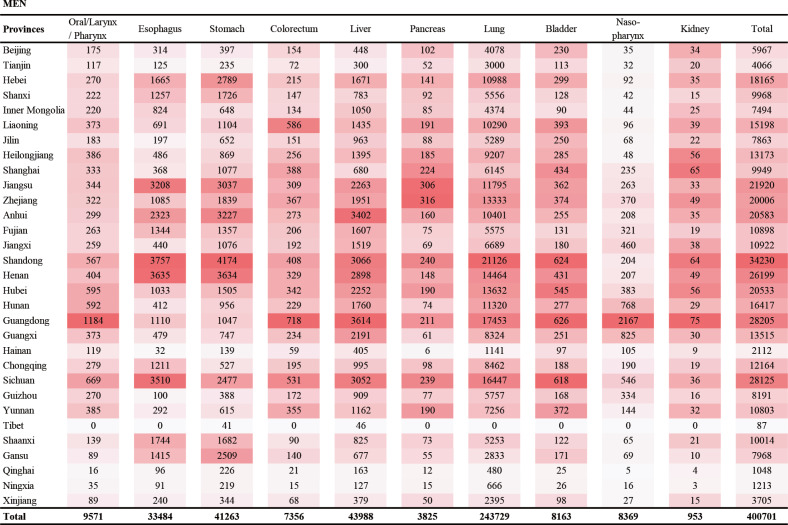
Heatmap of site-specific cancer deaths in the 31 provinces of mainland China in 2013 in men

**Figure 3:**
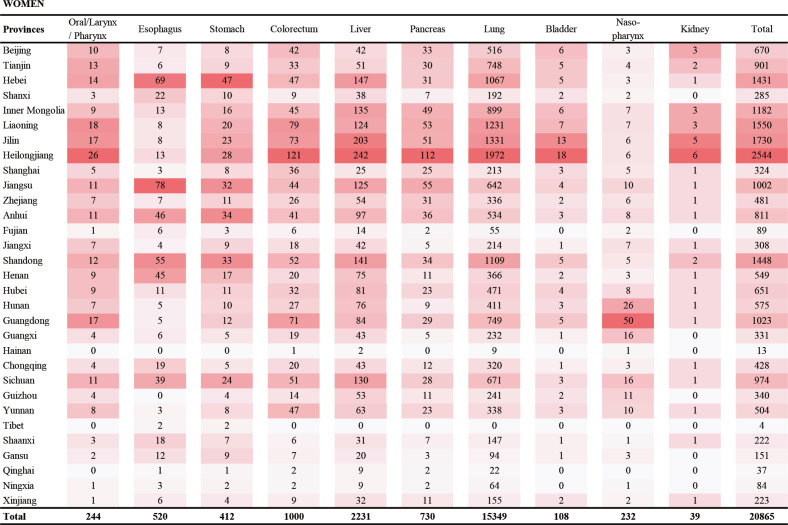
Heatmap of site-specific cancer deaths in the 31 provinces of mainland China in 2013 in women

We also present the site-specific smoking-attributable cancer deaths and the corresponding proportions in 31 provinces among men and women (Figures 2 and 3; and Supplementary file, Figures S2 and S3). Overall, cancers of lung and liver were the two main causes of smoking-related cancer deaths for both men and women. For men, stomach cancer was the third cause of smoking-related cancer deaths, followed by cancers of esophagus, mouth/larynx/pharynx, nasopharynx, bladder, colorectum, pancreas, and kidney. For women, colorectal cancer was the third cause of smoking-related cancer deaths, followed by cancers of pancreas, esophagus, stomach, mouth/larynx/pharynx, nasopharynx, bladder, and kidney. The number and proportion of site-specific smoking-attributable cancer deaths varied substantially across provinces.

## DISCUSSION

This study provides a systematic evidence-based assessment of province-specific smoking-attributable cancer burden in China. Smoking-attributable cancer mortality in men (PAF=29.34%; 95%CI: 19.21–42.31%) was substantially higher than that in women (PAF=2.61%; 95%CI: 1.01–4.31%), which is mainly explained by the higher smoking prevalence in men. Overall, smoking was responsible for approximately 20% in all cancer deaths in mainland China, but this proportion of smoking-attributable cancer deaths varied substantially across provinces.

Regional variation in PAFs can be primarily explained by differences in smoking prevalence, which has prevailed partly due to variations in tobacco control policies and programs, socioeconomic status and education, culture, and attitudes. Since China ratified the WHO Framework Convention on Tobacco Control in 2005, some metropolitan cities have adopted smoke-free laws prohibiting smoking in public areas. However, the implementation and compliance of smoke-free policy has shown large differences on the subnational level^23^. Tobacco control policies are heavily influenced by the tobacco industry in all provinces, and tobacco use prevalences are higher in the cities near where tobacco is grown, such as in the Yunnan and Guizhou provinces. However, higher smoking prevalences in the Northeast regions, with nearly no tobacco plantations, can be explained by low smoking cessation rates and low cost of manufactured cigarettes there^24^. Moreover, low socioeconomic status is considered to be associated with higher smoking prevalence and lower smoking cessation rates^25,26^. People with lower educational levels are less aware of the adverse effects of smoking. Generally, cigarettes are a popular ‘social currency’ in China, especially in the rural regions^27,28^.

The larger burden of smoking-related cancer deaths in men than in women most likely reflects the higher prevalence of smoking among men than in women, but there are large discrepancies in PAFs among women across provinces. PAFs among women living in the Southwest provinces were approximately 2–3%, which were lower than the national average, compared with that in men of Southwest China. Moreover, tobacco control policies and programmes appeared not to be well implemented in Northern China. The topmost 4 provinces (Jilin, Tianjin, Inner Mongolia, and Heilongjiang) had PAFs that were three-fold higher than the national average among women, and the top nine provinces with higher PAFs were all located north of the Yangtze River.

The order of site-specific cancer deaths in China has changed in the past decades^29^, but lung cancer have ranked as first in the smoking-attributable cancer deaths for both genders, consistent with the results from other developing countries^30^. The tobacco epidemic was responsible for the rapid increase in lung cancer mortality in recent decades^31^. However, in some provinces, we found that smoking contributed more deaths from cancers of the stomach and esophagus than liver cancer among men, which was driven by the higher mortality rates of stomach and esophageal cancers in these regions.

Our province-specific PAF estimates were comparable with those from the limited evidence available from previous studies in China based on similar methodology. In our study, estimates of provincial PAFs for smoking and cancer deaths in men were higher than the corresponding figures of a previous study^32^. This discrepancy can be primarily explained by different sources of smoking prevalence. Our study used the smoking prevalence in 1998 as the exposure rate, which was higher than the figures of 2002 used in the Xia et al.^32^ study, based on a different assumption of the latency time. Although the smoking definitions were similar in both studies, there were some differences in how PAF values were estimated. For smoking-related cancers, we included 10 cancer sites in our study versus 5 major cancer sites together with other minor sites in the Xia et al.^32^ study. In addition, the estimates of smoking attributable cancer mortality could be affected by the time fluctuation of cancer registered data and different sources of RRs. For most cancers, RRs used in our estimates were abstracted from the results of the Chinese population in a pooled analysis of 21 cohort studies in Asia, which were relatively lower than those in the Western population^33,34^, but higher than those in the Xia et al.^32^ study. In their study, RRs were taken from the China Kadoorie Biobank Study from 10 regions during 7 years of follow-up^35^, which might be underestimated due to the short period of follow-up. Regardless of the PAF disparities, the order of provincial PAFs was still comparable in the two studies (Supplementary file, Figure S4).

In China, smoking prevalence in men declined rapidly during the 1980s and 1990s. Nevertheless, the fraction of smoking-attributable cancer mortality increased slightly over time. The Liu et al.^36^ study in 1990 reported a fraction of 24.4% in men, but the corresponding figures were 28.0% and 32.7% in 2005 in the Gu et al.^12^ study and the Wang et al.^8^ study, respectively. There might be potential overestimation in relative risk for lung cancer sourced from limited Shanghai residents used in the Wang et al.^8^ study. Methodological differences might be a source of disparity, but potential transition in stages of smoking-related cancer might be a greater contributor. Moreover, our PAF estimates were comparable with the results from the Western populations^37^, while smoking prevalence was relatively higher in Chinese men^7,15,16^. In China, widespread tobacco smoking began several decades later than in Europe and North America, thus China was at an earlier stage of the tobacco epidemic compared with the developed countries^38^. In recent years, smoking prevalence has declined slowly in China due to enforcement of tobacco control policies, but still remains high, especially in men. The current smoke-free policy is still inadequate in reducing prevalence and affecting smoker’s behavior^39^. As the tobacco epidemic grows, smoking associated cancer deaths will be elevated due to the long latency time of smoking related cancers. More effective efforts in tobacco control, including increasing tobacco taxes and maintaining funding of anti-smoking campaigns, are needed to reduce the smoking-related cancer burden.

### Strengths and limitations

A strength of our study is the estimate of site-specific smoking attributable cancer mortality at the provincial level. However, our study has also several limitations and uncertainty. First, in our study, indirect smoking prevalence data were used due to lack of qualified age-specific data on provincial smoking prevalence and smoking prevalence based on self-reported results can be underestimated^40^, which could affect our estimates. Second, only 10 major types of smoking-related cancers were included in our study, and some other smoking-associated cancers were excluded due to the lack of reliable data, so that our PAF values might be underestimated. Third, secondhand smoking also plays a crucial role in the association of smoking and lung cancer, but it was not included in our study because it is difficult to quantify and there are no corresponding reliable provincial data based on latency time. However, in our previous study, we estimated that 11.1% of lung cancer deaths among non-smoking women were attributable to involuntary smoking from the spouse or at the workplace^10^, which is very comparable with that of the Xia et al.^32^ study (11.5%). Finally, in our study, we only considered smoking status as never or ever, and were not able to collect information on the type, starting age, amount and duration of smoking. However, using alternative definitions of smoking may not substantially alter the PAF estimates based on the Xia et al.^32^ study.

## CONCLUSIONS

Our study provides a systematic assessment of province-specific cancer burden of tobacco smoking in China in 2013. We found that smoking was responsible for nearly 20% of cancer deaths in the Chinese population in 2013, and that the proportion of smoking-attributable cancer deaths varies across provinces. Our findings provide strong evidence for more effective programs and innovative new strategies for local tobacco control to reduce the high burden of smoking-related cancers in the provinces of mainland China.
