# Supplementary material for: Province-specific smoking-attributable cancer mortality in China 2013
Source: Tob Induc Dis. 2020 Jun 1;18:49. doi: 10.18332/tid/122013 (PMC7291958; doi:10.18332/tid/122013)
Supplement: Supplementary file 1 [file TID-18-49-s1.pdf]

Supplementary Online Material

Figure S1. Rank of Smoking-attributable Cancer Mortality in 2013 in both sexes

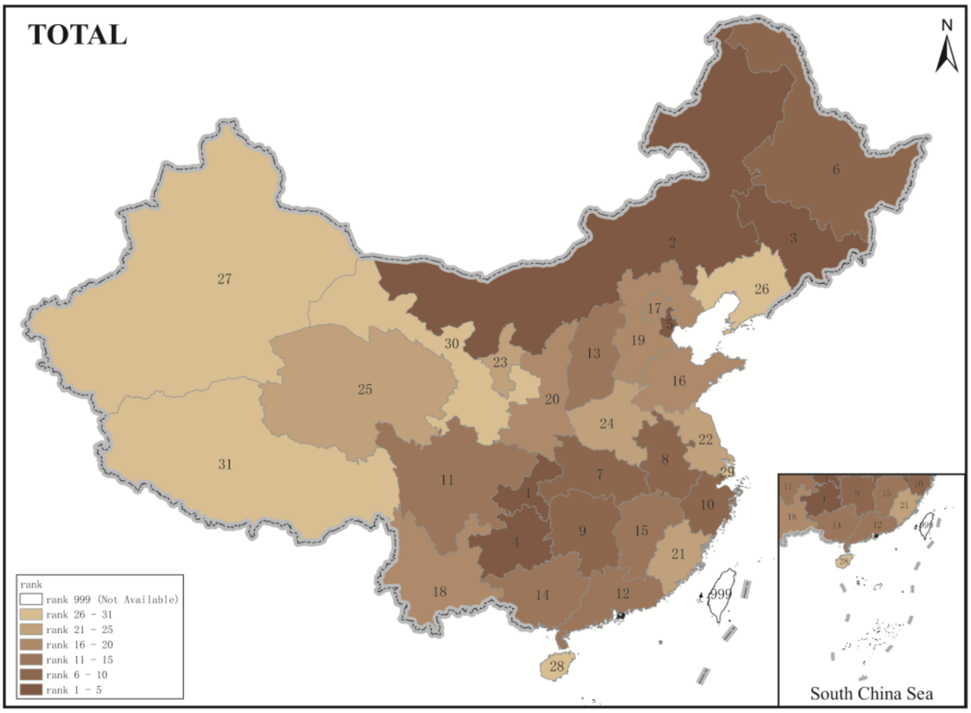

Figure S1. Rank of Smoking-atrributable Cancer Mortality in 2013 in both sexes

Figure S2. Proportions of site-specific cancer mortality in the 31 provinces of mainland China in 2013 in men

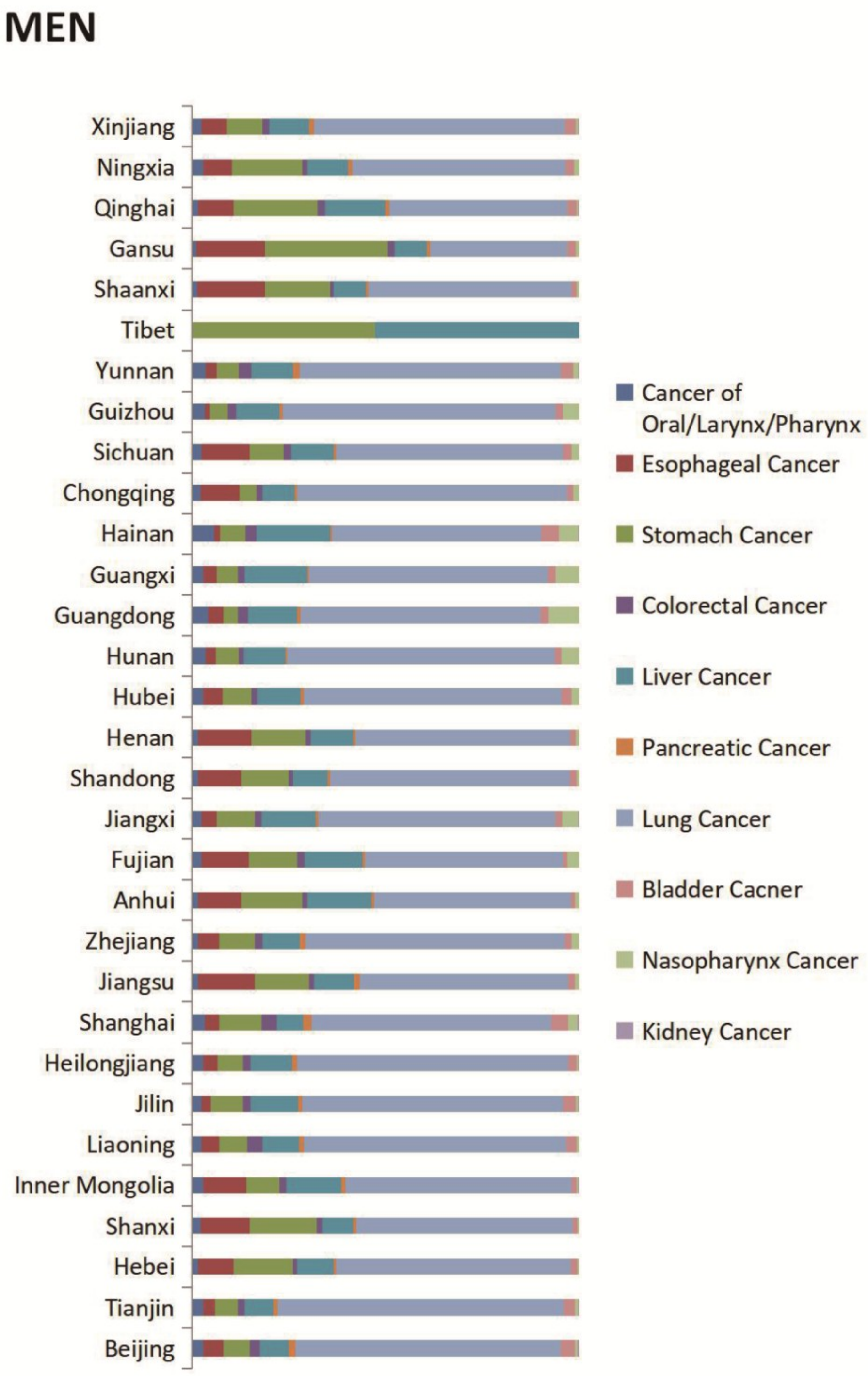

Figure S2. Proportions of site-specific cancer mortality in the 31 provinces of mainland China in 2013 in men

Figure S3. Proportions of site-specific cancer mortality in the 31 provinces of mainland China in 2013 in women

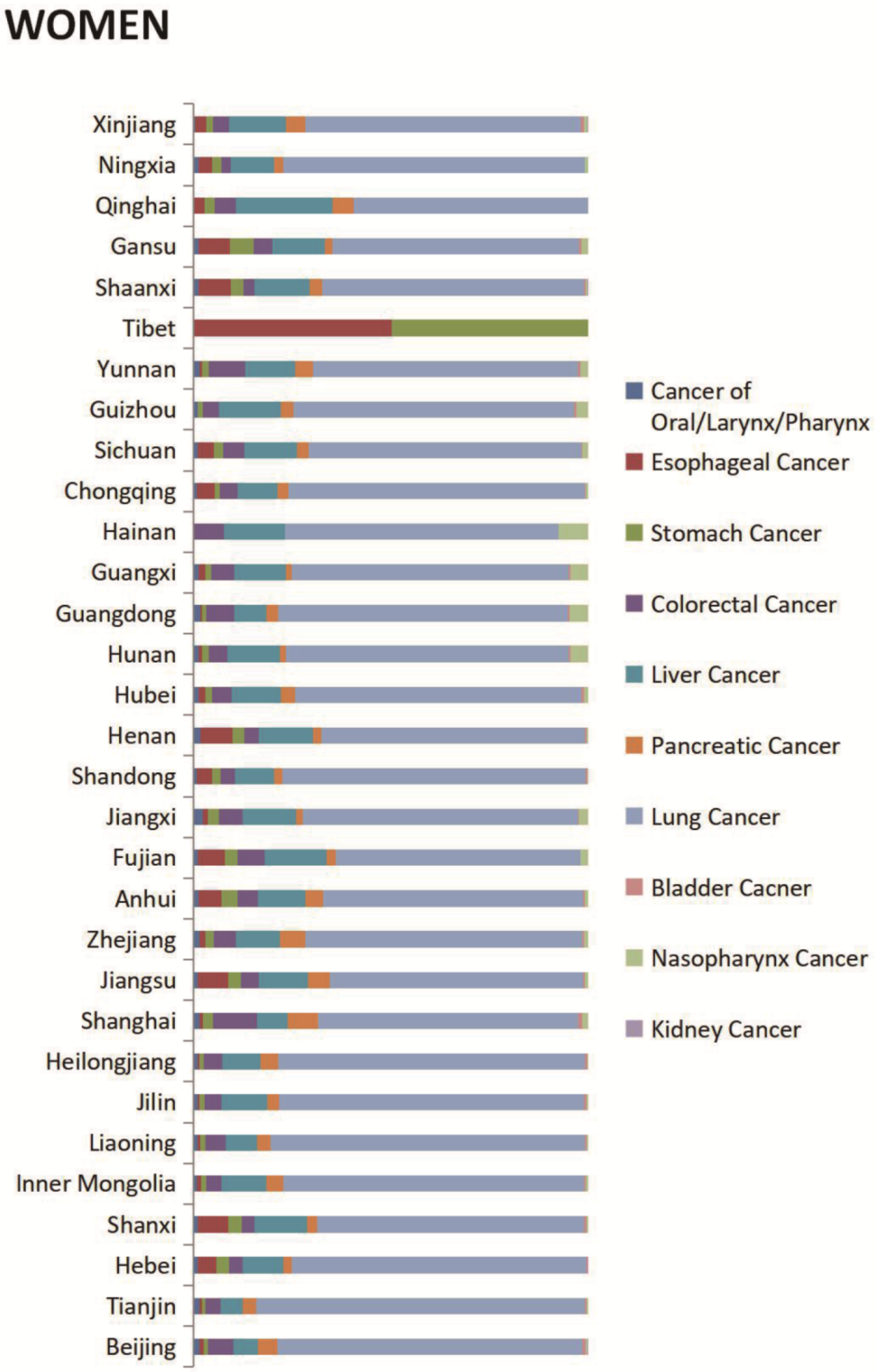

Figure S3. Proportions of site-specific cancer mortality in the 31 provinces of mainland China in 2013 in women

Figure S4. Comparison of provincial PAF order between our study and the previous study

	Men		Women	
	Our study	Xia's study	Our study	Xia's sudy
Beijing	16	22	5	9
Tianjin	6	7	2	2
Hebei	22	16	6	16
Shanxi	13	4	21	19
Inner Mongolia	7	9	3	4
Liaoning	21	13	8	6
Jilin	4	20	1	3
Heilongjian	9	18	4	1
Shanghai	26	25	25	27
Jiangsu	23	6	13	15
Zhejiang	9	14	24	22
Anhui	8	19	10	10
Fujian	24	11	30	26
Jiangxi	15	21	22	31
Shandong	18	8	14	12
Henan	17	15	28	29
Hubei	3	17	18	11
Hunan	5	3	16	14
Guangdong	12	10	18	21
Guangxi	19	29	23	17
Hainan	30	30	31	28
Chongqing	2	5	12	23
Sichuan	14	23	17	20
Guizhou	1	1	11	13
Yunnan	11	2	15	24
Tibet	31	26	27	5
Shaanxi	20	27	26	30
Gansu	29	12	29	18
Qinghai	27	24	20	25
Ningxia	25	28	7	8
Xinjiang	28	31	9	7

PAF min

PAF max

*rank of PAF here in Xia's study indicates to the rank of standardised PAF

Figure S4. Comparison of provincial PAF order between our study and the previous study

© 2020 Yu L. et al.
